# Biomarkers in *Trypanosoma cruzi*-Infected and Uninfected Individuals with Varying Severity of Cardiomyopathy in Santa Cruz, Bolivia

**DOI:** 10.1371/journal.pntd.0003227

**Published:** 2014-10-02

**Authors:** Emi E. Okamoto, Jacqueline E. Sherbuk, Eva H. Clark, Morgan A. Marks, Omar Gandarilla, Gerson Galdos-Cardenas, Angel Vasquez-Villar, Jeong Choi, Thomas C. Crawford, Rose Q., Antonio B. Fernandez, Rony Colanzi, Jorge Luis Flores-Franco, Robert H. Gilman, Caryn Bern, for the Chagas Disease Working Group in Bolivia and Peru

**Affiliations:** 1 New York University School of Medicine, New York, New York, United States of America; 2 Baylor College of Medicine, Houston, Texas, United States of America; 3 Johns Hopkins University Bloomberg School of Public Health, Baltimore, Maryland, United States of America; 4 Beth Israel Deaconess, Boston, Massachusetts, United States of America; 5 Hospital Nacional Guillermo Almenara Ingoyen, EsSalud, Lima, Peru; 6 University of Michigan School of Medicine, Ann Arbor, Michigan, United States of America; 7 VA Medical Center and University of Colorado School of Medicine, Denver, Colorado, United States of America; 8 Hartford Hospital, Hartford, Connecticut, United States of America; 9 Universidad Catolica Boliviana, Santa Cruz, Plurinational State of Bolivia; 10 University of California San Francisco, San Francisco, California, United States of America; Emory University, United States of America

## Abstract

**Background:**

Twenty to thirty percent of persons with *Trypanosoma cruzi* infection eventually develop cardiomyopathy. If an early indicator were to be identified and validated in longitudinal studies, this could enable treatment to be prioritized for those at highest risk. We evaluated cardiac and extracellular matrix remodeling markers across cardiac stages in *T. cruzi* infected (Tc+) and uninfected (Tc−) individuals.

**Methods:**

Participants were recruited in a public hospital in Santa Cruz, Bolivia and assigned cardiac severity stages by electrocardiogram and echocardiogram. BNP, NTproBNP, CKMB, troponin I, MMP-2, MMP-9, TIMP-1, TIMP-2, TGFb1, and TGFb2 were measured in specimens from 265 individuals using multiplex bead systems. Biomarker levels were compared between Tc+ and Tc− groups, and across cardiac stages. Receivers operating characteristic (ROC) curves were created; for markers with area under curve>0.60, logistic regression was performed.

**Results:**

Analyses stratified by cardiac stage showed no significant differences in biomarker levels by Tc infection status. Among Tc+ individuals, those with cardiac insufficiency had higher levels of BNP, NTproBNP, troponin I, MMP-2, TIMP-1, and TIMP-2 than those with normal ejection fraction and left ventricular diameter. No individual marker distinguished between the two earliest Tc+ stages, but in ROC-based analyses, MMP-2/MMP-9 ratio was significantly higher in those with than those without ECG abnormalities.

**Conclusions:**

BNP, NTproBNP, troponin I, MMP-2, TIMP-1, and TIMP-2 levels rose with increasing severity stage but did not distinguish between Chagas cardiomyopathy and other cardiomyopathies. Among Tc+ individuals without cardiac insufficiency, only the MMP-2/MMP-9 ratio differed between those with and without ECG changes.

## Author Summary

In Chagas disease, a parasitic disease found primarily in Central and South America, individuals are chronically infected with the parasite *Trypanosoma cruzi*. A few decades after initial infection, 20–30% of infected individuals will develop cardiac disease. If we were able to predict which individuals would progress to cardiac disease, treatment in these low resource areas could be targeted to those at highest risk. The ideal transition point to identify those at risk would be as individuals progress from a normal electrocardiogram to an abnormal electrocardiogram, the first step in the progression of cardiac disease. Previous studies have suggested a group of serum biomarkers able to differentiate between these stages of disease. However, our larger and more comprehensive study finds that none of the ten cardiac and remodeling biomarkers we tested are able to distinguish between healthy individuals and those with the earliest evidence of cardiac disease. We did find cardiac biomarkers to be elevated in those with severe cardiac disease as expected in both *T. cruzi* infected and uninfected individuals.

## Introduction

Chagas disease (American trypanosomiasis) is caused by the protozoan *Trypanosoma cruzi*. In the Western Hemisphere, an estimated 8 million individuals are currently infected with the parasite and the disease is responsible for four times more disability-adjusted life years lost than malaria [1], [2]. While Chagas disease traditionally affects rural communities, migration has resulted in its spread outside endemic areas [2]. In a hospital in Santa Cruz, the largest city in Bolivia, 60% of congestive heart failure and 79% of advanced heart failure cases were associated with *T. cruzi* infection [3].

Chagas cardiomyopathy (CCM) is the primary cause of morbidity and mortality in Chagas disease. Chagas disease begins with an acute phase lasting 4–8 weeks, followed by chronic infection and a long asymptomatic period. Of those infected, 20–30% will eventually progress to overt cardiac disease over decades [2]. A chronic inflammatory state due to the persistence of the parasite results in damage to the conduction system and myocardium [4]. Patients initially present with conduction defects such as bundle branch blocks [3] with later progression to high-grade atrioventricular block and complex ventricular arrhythmias [4] [5]. Advanced disease is characterized by progressive, often intractable congestive heart failure [2]. CCM is associated with more hospitalizations and shorter survival than dilated cardiomyopathy of other etiologies [6], [7]. In Bolivia and other resource-constrained settings, patients have limited access to anti-arrhythmic medications, pacemakers and implantable cardiac defibrillators, rendering heart block and arrhythmias more lethal than in developed countries [8].

Although antitrypanosomal treatment of children with chronic infection is now the standard of care throughout the Americas, adult treatment is still a matter of debate, because of the lack of an accepted test of cure and the high frequency of adverse effects with the only available drugs [1], [4], [9]. A non-randomized, non-blinded observational study suggested that treatment may decrease progression of CCM [10]. The BENEFIT trial (clinicaltrials.gov/NCT00123916) is currently underway to assess benznidazole efficacy to prevent progression in adults with early signs of CCM [11]. However, antitrypanosomal treatment will not reverse structural damage and is poorly tolerated in advanced CCM [4]. Thus, the optimal time to treat *T. cruzi*-infected (Tc+) individuals is prior to onset or during the earliest stage of CCM. To date, no marker has been shown to predict progression to CCM [4]. If a reliable early indicator were identified, treatment efforts could prioritize those with the highest likelihood of benefit.

Our primary aim was to evaluate candidate biomarkers across the clinical severity spectrum in Tc+ individuals. Our secondary aim was to compare Tc+ to uninfected (Tc−) individuals with and without heart failure to assess specificity of the markers for CCM. We evaluated three known cardiac structural markers. Brain Natriuretic Peptide (BNP) and N-terminal brain natriuretic peptide (NTproBNP) are released under myocardial wall stress and commonly used in the diagnosis and management of heart failure [12]. Troponins reflect ischemia and inflammation [13], [14]. Creatine kinase-MB (CKMB) is used as a measure of heart failure severity [15]. Elevated BNP, NTproBNP and Troponin I levels have been reported in CCM, but data for these markers in asymptomatic Chagas disease are sparse [16]–[20].

We also evaluated matrix metalloproteinase (MMP)-2, MMP-9, tissue inhibitor of metalloproteinase (TIMP)-1, TIMP-2, and transforming growth factor beta (TGFb)1 and TGFb2. Matrix metalloproteinases reflect extracellular matrix degradation and remodeling. TIMP-1 and TIMP-2 are inhibitors of MMP-9 and MMP-2, respectively [21]. An imbalance towards matrix degradation, with MMPs increased relative to TIMPs, is associated with ventricular dilatation and remodeling [13] and has been reported in heart failure [22]. Elevated MMP-2 and MMP-9 levels have been reported in CCM patients with ECG changes [23]. Elevated MMP-9 has also been associated with inflammation and CCM severity [24].

## Materials and Methods

### Ethics Statement

The study protocol was approved by the Institutional Review Boards of Universidad Catolica Boliviana (Santa Cruz, Bolivia), PRISMA (Lima, Peru), and Johns Hopkins Bloomberg School of Public Health (Baltimore, Maryland). All participants provided written informed consent.

### Study Population

**Figure 1:**
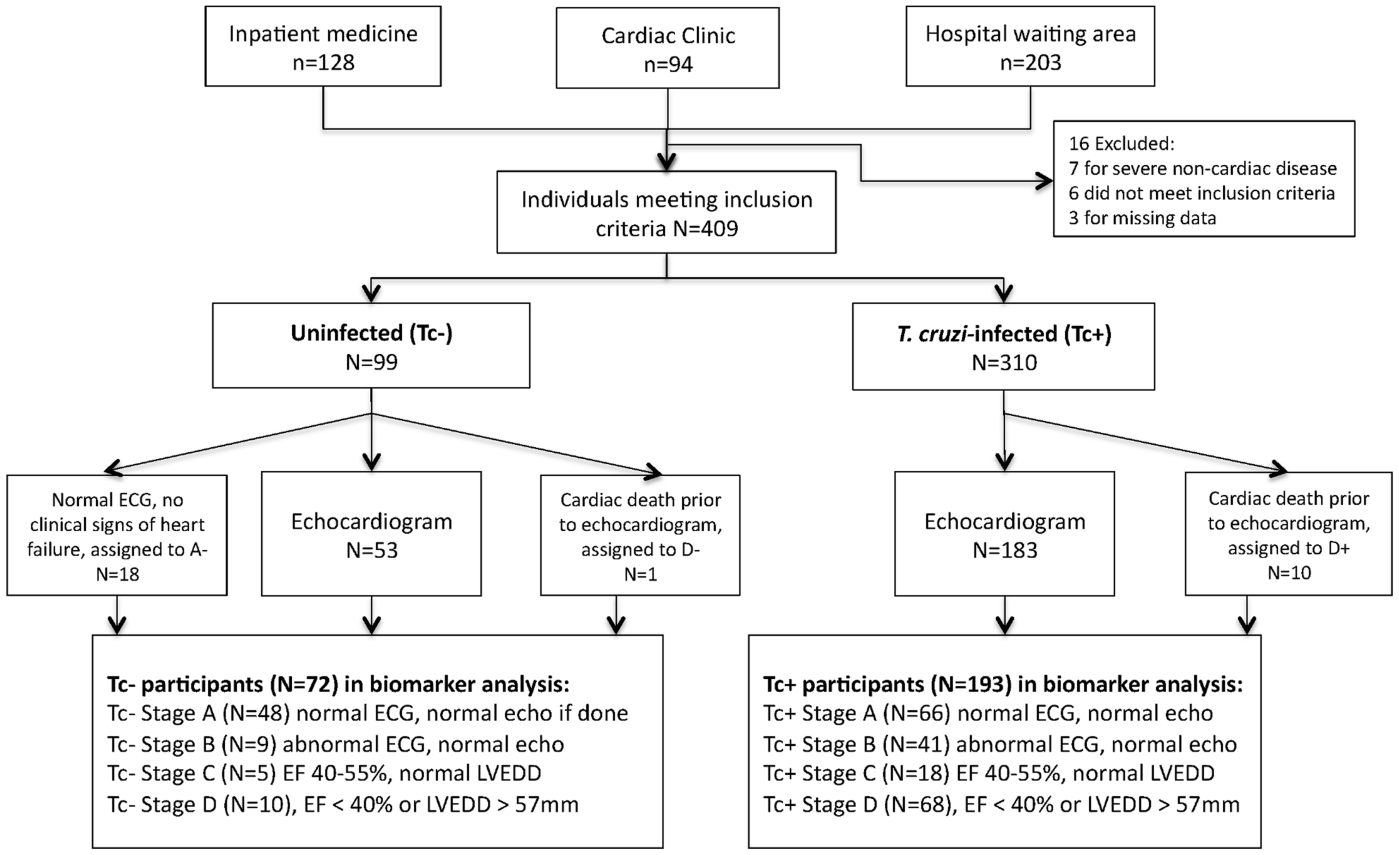
Study enrollment and staging classification.

Recruitment occurred from September 2012 to April 2013 at San Juan de Dios Hospital, the largest public hospital in Santa Cruz. Individuals 18 years or older were recruited from 3 sites: the internal medicine ward, the cardiac clinic, and the hospital waiting area (Figure 1). Ward patients with a history of cardiomyopathy from any cause were invited to participate. In the cardiac clinic, individuals with a history of Chagas disease or cardiomyopathy from any cause were recruited. The hospital waiting area included visitors, those awaiting a non-cardiac clinic appointment, and relatives of patients. These individuals were screened for eligibility and history of Chagas disease. Though *T. cruzi* status was unconfirmed at time of recruitment, suspected Tc+ individuals by history and risk factors were preferentially recruited. Uninfected participants were frequency-matched by age and sex to groups of Tc+ participants to yield comparable Tc+ and Tc− groups across the cardiac severity spectrum. Patients with severe non-cardiac disease (i.e. chronic renal failure, COPD, sepsis) were excluded, as were relatives of enrolled study members to avoid potential genetic influences on biomarkers. Based on published data on means and standard deviations of TGFb1 levels among Tc+ patients with and without cardiomyopathy [25], we calculated that a target sample size of 50 patients per Tc+ severity stage would provide 80% power and 95% confidence to detect differences between groups. No data were available for Tc+ vs Tc− patients; our target sample size for Tc− patients was 20 per stage.

### Data Collection

Epidemiologic data, socioeconomic factors, and medical history were collected using a structured questionnaire. Patients were assigned a New York Heart Association (NYHA) class based on symptoms, reported exercise tolerance, and history. An 18 cc whole blood specimen was collected, transported at room temperature to our Santa Cruz laboratory and centrifuged within 4 hours of collection. Electrocardiography was performed with Welch-Allyn portable ECG machine. A single cardiologist (AVV), blinded to Chagas status, performed 2D echocardiography with a Sonosite Micromaxx ultrasound. Echocardiograms were performed in three one-week blocks over the recruitment period on a subgroup of individuals (Figure 1). Left ventricular end diastolic diameter (LVEDD) was calculated in M-mode and ejection fraction (EF) in 2-dimensional mode. Segmental score was calculated based on the average wall motion of six areas, where a value of 1 signified normal motion and 2, 3, and 4 signified varying degrees of dysfunction (hypokinesis, akinesis, and dyskinesis, respectively).

### Laboratory Methods

Samples were centrifuged and separated into serum and clot. Sera were tested by an enzyme-linked immunoassay (depending on availability, either Wiener Recombinante or Chagatest, Wiener Laboratories, Rosario, Argentina) and indirect hemaagglutination test (Chagas Polychaco kit, Lemos Laboratories, Santiago del Estero, Argentina). For specimens with discordant results, the trypomastigote excreted-secreted antigens (TESA)-blot was performed following the published protocol [26]. *Trypanosoma cruzi* infection was considered confirmed based on positive results by at least 2 tests.

Serum aliquots were stored at −20C and transported to our laboratory in Lima, Peru for biomarker measurement by multiplex bead assays. Brain natriuretic protein, NTproBNP, CKMB, and troponin I were measured using the Milliplex Map Human Cardiovascular Disease Magnetic Bead Panel. Other biomarker levels were measured using Milliplex MAP Human MMP Magnetic Bead Panel 2, Milliplex Map Human TIMP Magnetic Bead Panel 1, and Milliplex MAP TGF-B1,2,3 Plex Magnetic Bead Panel (all kits from Millipore, Billerica, Massachusetts).

### Clinical Classification

Participants were assigned to severity stages based on ECG and echocardiogram results (Figure 1). Stages were based on a slight modification of the American College of Cardiology and American Heart Association heart failure classification system, previously used in CCM [3], [27], [28]. ECG criteria for stage B were based on characteristic abnormalities described in many studies of CCM, and comprised right bundle branch block (RBBB), left anterior fascicular block (LAFB), left bundle branch block (LBBB), incomplete RBBB (iRBBB), atrio-ventricular blocks (AVB), multifocal or paired ventricular premature beats, atrial fibrillation or flutter, or severe bradycardia (< = 50/min) [1], [29] with no structural changes by echocardiogram. Stage C was defined by mild to moderate systolic dysfunction (EF 40–54%) and stage D by severe systolic dysfunction (EF<40%) and/or severe left ventricular dilatation (LVEDD> = 57 mm). In marker analyses, stages C and D were combined, as both of these stages reflect the presence of cardiac insufficiency.

Not all individuals recruited underwent echocardiogram. To ensure echocardiograms were performed on individuals in all severity stages, participants were grouped based on history of heart failure and presence of ECG abnormalities. Within each group, individuals were randomly chosen for an echocardiogram appointment until all available appointments were filled. Eighteen uninfected individuals with normal ECG and no clinical signs of heart failure but without echocardiogram were classified as stage A. One Tc− individual and 10 Tc+ patients who died from cardiac disease prior to echocardiogram were included in stage D. Exclusion of the 29 subjects without echocardiogram from analysis did not significantly change our results; these subjects are included in our analyses.

### Statistical Analysis

Categorical variables are reported as percentages and compared using the Chi-square test, Fisher exact test, or ANOVA. Continuous variables are reported as mean and standard deviation or median and IQR based on normality as determined by Shapiro-Wilke test. Normally distributed variables were compared using a Student's t-test. Log-transformed biomarker levels and other non-parametric variables were compared using Wilcoxon rank sum. Biomarker values below the limit of detection (LOD) were assigned the LOD. Spearman's rank correlation was used to determine pairwise correlations of biomarkers with a Bonferroni correction for significance, and biomarker ratios were created for pairs with significant negative correlations. The Kruskal-Wallis test was used for multi-group comparisons. Receiver operating characteristic (ROC) curves were created and for those markers with area under the curve (AUC)s>0.60, univariate and multivariate logistic regression analyses were performed to determine an odds ratio (OR) and 95% confidence interval (CI) while adjusting for age and comorbidities. All statistical analyses were performed using Stata 12.0 with a two-tailed p< = 0.05 considered to be significant.

## Results

Tc+ participants were more likely than Tc− participants to report known risk factors for Chagas disease, including residence in a house with mud walls and in a house infested with the triatomine vector, and a family history of Chagas disease. Tc+ participants had lower educational attainment than Tc− participants, suggesting past poverty and/or remote residence. However, current socioeconomic indicators (having electricity, cell phone or refrigerator), and self-reported prevalence of hypertension and diabetes did not differ significantly by Tc status (Supplemental table S1).

Individuals with more severe disease were older, regardless of Tc status (Table 1). Thirty-seven percent of the population was overweight and 36% were obese. Among Tc+ patients, the mean BMI decreased significantly with severity. The most common ECG abnormalities among Tc+ patients were atrial fibrillation/flutter, bradycardia (heart rate <50 per minute), 1^st^ degree AV block, RBBB, iRBBB, LAFB, LBBB and combined LAFB/RBBB. Atrial fibrillation/flutter, bradycardia and any complete bundle branch block were significantly more common among Tc+ than Tc− patients (supplemental Table S2). Eleven Tc+ and 4 Tc−patients had pacemakers at the time of evaluation. Four additional Tc+ patients (ages 31, 39, 57, and 55) were found to have 3^rd^ degree AV-block on their study ECG; three had a pacemaker implanted during their admission while one declined. The mean EF in Tc+ stage C and D was 47.8 and 27.1, and average LVEDD in Tc+ stage D was 61.0 mm. Echocardiogram findings did not differ significantly between Tc+ and Tc− individuals in the same severity stage. Segmental wall motion scores increased with stage, regardless of Tc status.

**Table 1:** Demographic, electrocardiographic, and echocardiographic findings by stage in *T. cruzi*-infected (Tc+) and uninfected (Tc−) individuals.

	Tc+ Stage A	Tc− Stage A	Tc+ Stage B	Tc− Stage B	Tc+ Stage C	Tc− Stage C	Tc+ Stage D	Tc− Stage D
	N = 66	N = 48	N = 41	N = 9	N = 18	N = 5	N = 68	N = 10
	N (%)	N (%)	N (%)	N (%)	N (%)	N (%)	N (%)	N (%)
Male Sex	29 (43.9)	18 (37.5)	19 (46.3)	4 (44.4)	8 (44.4)	2 (40.0)	43 (63.2)	8 (80)
Age in years, mean (SD)	55.6 (12.7)	53.2 (12.8)	58.2 (12.7)	53.8 (15.6)	59.4 (12.6)	51.4 (13.0)	60.8 (11.6)	60.8 (14.3)
**Recruitment Site**								
Inpatient	5 (7.6)	3 (6.3)	8 (19.5)	3 (33.3)	10 (55.6)	3 (60.0)	48 (70.6)	8 (80)
Cardiology Clinic	15 (22.7)	8 (16.7)	15 (36.6)	3 (33.3)	4 (22.2)	2 (40.0)	16 (23.5)	2 (20)
Hospital Visitors	46 (69.7)	37 (77.1)	18 (43.9)	3 (33.3)	4 (22.2)	0 (0)	4 (5.9)	0 (0)
**Self Reported History of:**								
Hypertension	31 (47.0)	14 (29.2)	21 (51.2)	7 (77.8)	8 (44.4)	3 (60)	32 (47.1)	7 (70)
Diabetes	12 (18.2)	3 (6.3)	3 (7.3)	1 (11.1)	4 (22.2)	0 (0)	7 (10.3)	4 (40)
Coronary Artery Disease	7 (10.6)	1 (2.1)	6 (14.6)	0 (0)	4 (22.2)	1 (20)	12 (17.7)	5 (50)
Stroke	3 (4.6)	2 (4.2)	2 (4.9)	1 (11.1)	1 (5.6)	0	8 (11.8)	1 (10)
Pacemaker	0 (0)	0 (0)	3 (7.3)	3 (33.3)	1 (5.6)	1 (20)	7 (10.3)	0 (0)
BMI in kg/m2, mean (SD)	30.1 (5.4)	28.5 (6.1)	29.3 (5.8)	28.6 (6.3)	27.8 (7.6)	26.2 (6.3)	26.1 (4.6)	26.7 (5.3)
**ECG**								
HR in bpm, median (IQR)	66 (63,75)	69 (63,75)	**61 (57,67)**	**71 (61,75)**	68 (65, 92)	87 (60,98)	71 (63, 87)	75 (69,86)
PR Interval in ms, median(IQR)	157 (147, 168)	150 (146,174)	194 (168,220)	167.5 (155,177)	159 (150, 183)	153 (137,200)	164 (150, 187)	173.5 (157,212)
QRS Interval in ms, median(IQR)	95 (89, 107)	90.5 (83,101)	109 (95, 150)	103 (98,146)	115 (88, 140)	106.5 (102,152)	121 (100, 162)	141.5 (137,164)
Chagas-associated ECG changes	0 (0)	0 (0)	41 (100)	9 (100)	11 (61.1)	3 (60)	57 (83.8)	7 (70)
Bradycardia (HR< = 50)	0 (0)	0 (0)	7 (17.1)	0 (0)	1 (5.6)	0 (0)	7 (10.3)	0 (0)
Number of PVCs, mean (SD)	0.03 (0.2)	0 (0)	0.38 (1.4)	0.33 (1)	0.17 (0.4)	0.2 (0.1)	0.54 (1.2)	0.8 (1.6)
Multiple PVCs (>1)	0 (0)	0 (0)	4 (9.8)	1 (11)	0 (0)	0 (0)	11 (16.2)	2 (20)
Atrial Fibrillation	0 (0)	0 (0)	2 (4.9)	1 (11)	2 (11.1)	1 (20)	21 (30.9)	2 (20)
1st degree AV block	0 (0)	0 (0)	19 (46.3)	1 (11)	2 (11.1)	0 (0)	7 (10.3)	3 (30)
2nd degree AV block - Mobitz 1	0 (0)	0 (0)	0 (0)	0 (0)	1 (5.6)	0 (0)	0 (0)	0 (0)
3rd degree AV block	0 (0)	0 (0)	1 (2.4)	1 (11)	1 (5.6)	0 (0)	2 (2.9)	0 (0)
RBBB	0 (0)	0 (0)	9 (22.0)	2 (22)	2 (11.1)	0 (0)	11 (16.2)	1 (10)
Incomplete RBBB	0 (0)	0 (0)	7 (17.1)	1 (11)	2 (11.1)	0 (0)	2 (2.9)	1 (10)
LAFB	0 (0)	0 (0)	3 (7.3)	2 (22)	2 (11.1)	1 (20)	6 (8.8)	2 (20)
LBBB	0 (0)	0 (0)	3 (7.3)	1 (11)	2 (11.1)	0 (0)	7 (10.3)	1 (10)
Bifascicluar - LAFB and RBBB	0 (0)	0 (0)	5 (12.2)	0 (0)	0 (0)	0 (0)	4 (5.9)	1 (10)
**Echocardiography** *								
EF in percent, mean (SD)	62.0 (2.5)	62.3 (2.9)	61.3 (2.7)	61.7 (2.5)	47.8 (3.5)	50 (0)	27.1 (10.9)	24.4 (5.8)
LVEDD in mm, mean (SD)	43.4 (6.0)	42.9 (4.2)	45.7 (5.4)	45.0 (5.0)	46.5 (6.8)	43.7 (5.9)	61.0 (8.4)	55.9 (9.3)
Segmental Score, median (IQR)	1.0 (1.0–1.0)	1.0 (1.0–1.0)	1.0 (1.0–1.0)	1.0 (1.0–1.0)	1.25 (1.0–1.3)	1.25 (1.25–1.32)	2.16 (1.6–2.4)	1.9 (1.7–2.1)
Segmental Score>1	0 (0)	2 (6.7)	4 (9.8)	0 (0)	13 (72.2)	5 (100)	58 (100)	8 (88.9)

Bolded values represent a significant difference between Tc+ or Tc− individuals at a p<0.05.

*Not all individuals had echocardiography performed; Ns shown in Figure 1.

bpm = beats per minute, ms = millisecond, AV = atrio-ventricular, RBBB = right bundle branch block, LAFB = left anterior fascicular block, LBBB = left bundle branch block, EF = ejection fraction. LVEDD = left ventricular end diastolic diameter. na = not applicable.

In analyses stratified by severity stage, TGFb2 was significantly higher in Tc− than Tc+ stage A; no other biomarker level showed significant differences by *T. cruzi* status within the severity stage (Table 2). Both Tc+ and Tc− individuals showed progressive elevations in NTproBNP, BNP, and troponin I levels with increasing severity of cardiomyopathy. Infected individuals also showed progressively higher levels of MMP-2, TIMP-1, and TIMP-2 with increasing cardiac severity stage. Differences between marker levels for stage CD vs A and CD vs B were statistically significant (Table S3). Uninfected patients in stage CD also had significantly higher levels of CKMB compared to Tc− stage A or B. No significant differences were seen in biomarker levels between stages A and B, regardless of infection status.

**Table 2:** Comparison of biomarkers and biomarker ratios in *T. cruzi*-infected (Tc+) vs uninfected (Tc−) subjects by stage.

	Tc+ Stage A	Tc− Stage A	Tc+ Stage B	Tc− Stage B	Tc+ Stage CD	Tc− Stage CD
	N = 66	N = 48	N = 41	N = 9	N = 86	N = 15
**Biomarker** (pg/ml)						
BNP						
Median (IQR)	74 (5–143)	60 (5–147)	60 (5–161)	85 (65–260)	150 (43–333)	126 (19–193)
NTproBNP						
Median (IQR)	181 (121–321)	135 (87–233)	175 (124–296)	218 (153–621)	489 (263–981)	337 (279–567)
CKMB						
Median (IQR)	3957(2697–5911)	3532(2518–5639)	4158 (2974–5748)	4681(3174–4865)	4693(3011–7615)	5845 (4308–12549)
Troponin I						
Median (IQR)	42 (42–368)	42 (42–296)	151 (42–413)	105 (42–871)	240 (42–856)	399 (78–2195)
MMP-2						
Median (IQR)	79842 (68560–96981)	81116 (74563–92850)	81829 (70670–105747)’	101842 (83946–111654)	98821 (75549–139488)	90999 (78964–136924)
MMP-9						
Median (IQR)	195508 (152132–303014)	188300 (150727–239969)	176644 (131517–251436)	234599 (143093–330267)	166872 (113575–303348)	168461 (114684–319778)
TIMP-1						
Median (IQR)	147458 (123524–188501)	148468 (119882–200281)	145538 (122239–184633)	149220 (131043–205615)	182433 (131095–231587)	146117 (126184–227818)
TIMP-2						
Median (IQR)	**78493 (71547–96908)**	**88002 (76403–131667)**	78770 (70988–92332)	104752 (87134–113247)	98220 (71187–117996)	90351 (71597–99751)
TGFB1						
Median (IQR)	30336 (24817–35773)	32325 (26440–41304)	28812 (22058–32456)	30061 (28318–30968)	26861 (20592–34694)	25414 (24143–33194)
TGFB2						
Median (IQR)	**1163 (529–1840)**	**1641 (992–2147)**	1177 (440–1861)	1519(1096–1752)	1100 (605–1701)	883 (575–1518)
**Biomarker Ratios**						
MMP-2/MMP-9						
Median (IQR)	0.39 (0.28–0.54)	0.44 (0.30–0.65)	0.49 (0.35–0.72)	0.48 (0.38–0.59)	0.62 (0.30–1.16)	0.83 (0.43–1.05)
MMP-2/TGFB1						
Median (IQR)	2.7 (1.9–3.9)	2.6 (2.0–3.3)	3.0 (2.2–4.8)	3.3 (2.8–3.9)	4.1 (2.7–5.7)	3.1 (2.4–3.6)
MMP-2/TIMP-2						
Median (IQR)	1.04 (0.81–1.22)	0.93 (0.73–1.08)	1.08 (0.91–1.27)	0.92 (0.78–1.21)	1.11 (0.88–1.41)	1.04 (0.98–1.12)
MMP-9/TIMP-1						
Median (IQR)	1.41 (1.02–2.37)	1.27 (0.83–1.73)	1.22 (0.78–1.81)	1.37 (0.60–1.79)	0.93 (0.58–1.82)	1.04 (0.63–2.31)

Bolded values represent a significant difference between Tc+ or Tc− individuals at a p<0.05.

Receiver operating characteristic curves were generated for Tc+ stages CD versus Tc+ stages AB (Table 3 and Figure S1). Curves for BNP, NTproBNP, troponin I, MMP-2, TIMP-1 and TIMP-2 had AUCs>0.60 and were included in the regression analyses. Two ratios, MMP-2/MMP-9, and MMP-2/TGFb1, did not show significant differences across groups in the Kruskal-Wallis analysis, but showed AUCs of 0.61 and 0.65, respectively, and also entered the regression analyses. The ROC curve for NTproBNP had the highest AUC of 0.78; the cut point showed sensitivity and specificity of 72.9 and 72.6, respectively. All biomarkers and both biomarker ratios selected by ROC exhibited significance in univariate and multivariate analyses comparing Tc+ stages AB to CD (Table 4). Among models comparing Tc+ stage A to Tc+ stage B, only MMP-2/MMP-9 ratio showed a significant association with an OR (95% CI) of 3.24 (1.44–7.30).

**Table 3:** Results of ROC analysis of selected markers for discrimination between Stage AB and CD among *T. cruzi*-infected individuals.

Biomarker	AUC (95% CI)	Selected cut-point	Sensitivity	Specificity
BNP	0.65 (0.57–0.73)	103.64 pg/ml	60.0%	60.4%
NTproBNP	0.78 (0.71–0.85)	287.83 pg/ml	72.9%	72.6%
Troponin I	0.63 (0.55–0.70)	174.97 pg/ml	61.2%	62.3%
MMP-2	0.65 (0.57–0.73)	93311 pg/ml	60.2%	65.4%
TIMP-1	0.62 (0.53–0.71)	154120 pg/ml	66.2%	56.7%
TIMP-2	0.62 (0.53–0.71)	88677 pg/ml	63.4%	67.8%
**Biomarker Ratios**				
MMP-2/MMP-9	0.61 (0.53–0.69)	0.45 pg/ml	61.5%	54.2%
MMP-2/TGFB1	0.65 (0.57–0.73)	3.37 pg/ml	60.9%	65.1%

**Table 4:** Logistic regression comparing biomakers and biomarker ratios by cardiac stage among *T-cruzi* infected (Tc+) individuals. Cutoffs were determined by ROC analysis.

				AB vs CD			
	N(%)			Univariate		Multivariate*	
	Stage A	Stage B	Stage CD	OR (95% CI)	p-value	OR (95% CI)	p-value
**Biomarker (pg/ml)**							
BNP							
<103.64 pg/ml	29 (61.7)	24 (60.0)	34 (40.0)	1.0		1.0	
≥103.64 pg/ml	18 (38.3)	16 (40.0)	51 (60.0)	2.29 (1.28–4.09)	0.005	2.29 (1.26–4.16)	0.004
NTproBNP							
<287.83 pg/ml	40 (85.1)	30 (75.0)	23 (27.1)	1.0		1.0	
≥287.83 pg/ml	7 (14.9)	10 (25.0)	62 (72.9)	7.16 (3.77–13.59)	<0.001	7.14 (3.68–13.83)	<0.001
Troponin I							
<174.97 pg/ml	32 (68.1)	23 (57.5)	33 (38.8)	1.0		1.0	
≥174.97 pg/ml	15 (31.9)	17 (42.5)	52 (61.2)	2.60 (1.44–4.68)	0.001	2.48 (1.36–4.51)	0.003
MMP-2							
<93311 pg/ml	36 (75.0)	25 (61.0)	33 (39.8)	1.0		1.0	
≥93311 pg/ml	12 (25.0)	16 (39.0)	50 (60.2)	2.87 (1.58–5.19)	0.001	2.70 (1.47–4.98)	0.001
TIMP-1							
<154120 pg/ml	24 (54.5)	23 (62.2)	24 (33.8)	1.0		1.0	
≥154120 pg/ml	20 (45.5)	14 (37.8)	47 (66.2)	2.56 (1.34–4.88)	0.004	2.38 (1.23–4.59)	0.010
TIMP-2							
<88677 pg/ml	22 (0.5)	27 (73.0)	26 (36.6)	1.0		1.0	
≥88677 pg/ml	22 (0.5)	10 (27.0)	45 (63.4)	3.64 (1.89–7.01)	<0.001	3.57 (1.83–6.97)	<0.001
**Biomarker Ratio**							
MMP-2/MMP-9							
<0.45 pg/ml	26 (54.2)	15 (36.6)	32 (38.6)	1.0		1.0	
≥0.45 pg/ml	22 (45.8)	26 (63.4)	51 (61.4)	1.88 (1.05–3.38)	0.033	2.05 (1.10–3.83)	0.021
MMP-2/TGFB1							
<3.37 pg/ml	36 (0.75)	24 (64.9)	32 (39.0)	1.0		1.0	
≥3.37 pg/ml	12 (0.25)	13 (35.1)	50 (61.0)	2.91 (1.59–5.30)	<0.001	3.02 (1.62–5.63)	<0.001

*Multivariate analyses adjusted for age, sex, and presence of comorbidities (hypertension, diabetes, and coronary artery disease).

Among Tc+ patients, BNP, NTproBNP, Troponin I and MMP-2 showed significant positive correlations with severity stage (Figure S2). Similar correlations were seen among Tc− individuals, although limited by the smaller sample size. Correlation analysis of marker pairs was also performed among Tc+ individuals stratified by stage (Figure S3). Three marker pairs were significantly correlated within each stage: MMP-2 and TIMP-2, TGFb1 and TGFb2, and TIMP-1 and TIMP-2. Among Tc+ stage CD individuals, BNP, NTproBNP, and MMP-2 were also significantly correlated.

## Discussion

Our study is one of the most comprehensive analyses of biomarkers in Chagas cardiomyopathy to date, having examined 10 different serum molecules in 193 *T. cruzi*-infected patients across the CCM severity spectrum, as well as 72 uninfected patients. We showed significant elevations in BNP, NTproBNP, Troponin I, MMP-2, TIMP-1 and TIMP-2 among Tc+ individuals with cardiomyopathy compared to those without heart disease, findings consistent with previous studies [18]–[20], [22], [23]. However, these markers were not specific to Chagas cardiomyopathy and showed no differences by infection status in analyses stratified by cardiac severity stage. While no individual marker distinguished between infected individuals in stage A versus stage B, the MMP-2/MMP-9 ratio showed a significant association with escalating severity stage in several analyses. This transition from stage A (normal ECG) to stage B (ECG changes only) is the point most important to identify in order to detect those likely to progress to cardiomyopathy early enough to maximize the impact of antitrypanosomal treatment.

Our severity classification system was based on those described in the literature, and underscores the similarities between Chagas cardiomyopathy and dilated cardiomyopathy of other etiologies [1], [28]. Some recent analyses use segmental wall motion score as an additional grouping criterion, based on the hypothesis that these findings are among the earliest changes in CCM [28], [30]. However, in our data, the segmental score correlated well with Tc+ severity classes based on ECG changes, LVEDD and LVEF, suggesting that these measures are sufficient to classify individuals. The prevalence of segmental abnormalities was similar to that previously reported and comparable associations were also found for Tc− individuals [28].

Brain Natriuretic Peptide, NTproBNP and troponin, all known cardiac biomarkers, showed strong associations with dilated cardiomyopathy in both infected and uninfected participants [14]. Our results are consistent with previous studies of these markers in CCM [18]–[20], [31]. Perhaps because of its long half-life, NTproBNP showed the strongest association with cardiac disease [24]. Elevated troponin I has been reported previously in CCM compared to Tc+ stage A, and Tc+ compared to Tc− stage A [20]. Our study also demonstrated higher troponin in Tc+ stage CD versus Tc+ stage A, but we found no difference between infected and uninfected participants without heart disease (stage A).

We found that markers of tissue remodeling were up-regulated in Chagas cardiomyopathy [22]–[24]. Although MMP-2 levels did not differ significantly between Tc+ stage A vs B, the level of MMP-2 rose progressively across the severity classes in infected subjects [22]–[24]. Levels of TIMP-1 and TIMP-2 were also higher in Tc+ stage CD compared to Tc+ stages A and B. Like other cardiomyopathies, Chagas heart disease involves substantial tissue remodeling and progressive fibrosis; the changes seen in MMP and TIMP levels are a reflection of the severity of these processes.

Other biomarkers that showed promise in the literature did not have discriminatory power in our data. Transforming growth factor beta is a regulator of the inflammatory immune response, and is involved in *T. cruzi* invasion and cardiac pathogenesis in experimental animal models [32]. Unlike animal models and one earlier human study [25], we did not detect a significant difference in TGFb1 levels in patients with Chagas heart disease compared to those with the indeterminate form. Data from a smaller study by our group in Bolivia identified higher levels of TGFb1 and TGFb2 among infected individuals in stage B compared to stage A (Clark et al, unpublished data). Interestingly, both of our Bolivian studies showed significantly lower TGFb2 levels in Tc+ compared to Tc− stage A (p = 0.01), but our data are not currently sufficient to assess whether this finding has biological significance. Longitudinal studies would provide a more rigorous evaluation of TGFb levels as potential biomarkers in Chagas heart disease.

We found higher levels of MMP-9 than MMP-2, consistent with a prior study of CCM [24], but also found progressively higher MMP-2/MMP-9 ratio with increasing severity stages. Our findings are in conflict with a recent article reporting that MMP-9 levels increased relative to MMP-2 in severe CCM [24]. Matrix metalloproteinase-9 is released by inflammatory cells and associated with inflammatory myocarditis, suggesting that the ratio may reflect the balance of inflammation to fibrosis [22], [23], [33], [34]. If so, the increasing ratio in our data is consistent with a progression from early inflammation to late fibrosis. We also investigated the ratio of MMP-2 to TGFb1. MMPs and TGFb1 are reported to participate in a positive feedback loop in the myocardium [35]–[37]. In our data, the MMP-2/TGFb1 ratio increased significantly with increasing CCM severity, again possibly reflective of increasing fibrosis late in the disease.

The most salient limitation of our study is its cross-sectional design, which restricts our analyses to associations with disease severity. The gold-standard outcome, progression to more severe disease, can only be assessed in a longitudinal cohort. A cohort would also provide serial data to detect intra-individual changes in biomarker levels that may signal disease progression. The current study was not population-based, and therefore cannot give insight into the disease burden in the general population. We excluded patients with severe non-cardiac diseases, but co-morbidities such as hypertension, diabetes and obesity may have altered biomarker levels. Our severe cardiac disease groups were older, reflecting the natural history of cardiac disease.

In conclusion, our analysis provides extensive data on a range of biomarkers in *T. cruzi*-infected and uninfected individuals across the cardiac severity spectrum. Known markers of cardiac structural damage, BNP, NTproBNP and troponin, showed the expected associations in both infected and uninfected participants. Ratios of MMP-2/MMP-9 and MMP-2/TGFb1 also showed progressive associations across the severity spectrum in CCM. We were unable to identify an individual biomarker associated with the early changes of CCM. Potential biomarkers continue to be investigated through methods ranging from mass spectrometry [38] to serum proteonomics profiling [39], including hypercoagulability markers [40] and apolipoproteins [38]. Test panels for potential markers predictive of progression to CCM could further be expanded to investigate these new proteins. Given the growing burden of non-communicable diseases, it will also be important to analyze interactions of CCM with hypertension, diabetes, and obesity that increasingly affect Latin American populations [3].

## Supporting Information

Checklist S1 STROBE checklist.
(DOCX)

Figure S1 Receiver operating characteristic (ROC) curves for selected biomarkers. The curves compare *T-cruzi* infected individuals in stage AB versus stage CD. Diagonal reference line indicates an AUC value of 0.5.
(DOCX)

Figure S2 Correlation between stage and biomarker levels, stratified by *T. cruzi* infection status. 2a: *T. cruzi*-infected individuals. 2b: Uninfected individuals. Rho values are shown. Significant correlations (p<0.05) are shaded gray (positive correlation) and black (negative correlation).
(DOCX)

Figure S3 Correlation between biomarker levels among *T. cruzi*-infected individuals stratified by stage. 3a: Stage AB. 3b: Stage CD. Rho values are shown. Significant positive correlations (p<0.05) are shaded gray. No significant negative correlations were observed.
(DOCX)

Table S1 Basic characteristics and risk factors by *T. cruzi* infection status in all individuals meeting inclusion criteria.
(DOCX)

Table S2 ECG findings suggestive of Chagas disease by *T. cruzi* infection status for individuals included in biomarker analysis.
(DOCX)

Table S3 Comparisons of biomarkers and biomarker ratios by stage within *T. cruzi*-infected (Tc+) and uninfected (Tc−) groups.
(DOCX)
