# Correction: Biomarkers in *Trypanosoma cruzi*-Infected and Uninfected Individuals with Varying Severity of Cardiomyopathy in Santa Cruz, Bolivia

**DOI:** 10.1371/journal.pntd.0003362

**Published:** 2014-10-30

**Authors:** The PLOS Neglected Tropical Diseases Staff

The tenth author’s last name is omitted. The correct name is: Rose Q. Do.
