# Supplementary material for: Biomarkers in Trypanosoma cruzi-Infected and Uninfected Individuals with Varying Severity of Cardiomyopathy in Santa Cruz, Bolivia
Source: PLoS Negl Trop Dis. 2014 Oct 2;8(10):e3227. doi: 10.1371/journal.pntd.0003227 (PMC4183477; doi:10.1371/journal.pntd.0003227)
Supplement: Figure S1 — Receiver operating characteristic (ROC) curves for selected biomarkers. The curves compare T-cruzi infected individuals in stage AB versus stage CD. Diagonal reference line indicates an AUC value of 0.5. (DOCX) [file pntd.0003227.s002.docx]

**Figure S1: Receiver operating characteristic (ROC) curves for selected biomarkers. The curves compare *T-cruzi* infected individuals in stage AB versus CD. Diagonal reference line indicates an AUC value of 0.5.**

2a. BNP 2e. TIMP-1

2b. NTproBNP

2c. Troponin I

2d. MMP-2

2f. TIMP-2

2g. Ratio MMP2/MMP9

2h. Ratio MMP2/TGFB1
