# Supplementary material for: Biomarkers in Trypanosoma cruzi-Infected and Uninfected Individuals with Varying Severity of Cardiomyopathy in Santa Cruz, Bolivia
Source: PLoS Negl Trop Dis. 2014 Oct 2;8(10):e3227. doi: 10.1371/journal.pntd.0003227 (PMC4183477; doi:10.1371/journal.pntd.0003227)
Supplement: Figure S2 — Correlation between stage and biomarker levels, stratified by T. cruzi infection status. 2a: T. cruzi-infected individuals. 2b: Uninfected individuals. Rho values are shown. Significant correlations (p<0.05) are shaded gray (positive correlation) and black (negative correlation). (DOCX) [file pntd.0003227.s003.docx]

**Figure S2: Correlation between stage and biomarker levels, stratified by *T. cruzi* infection status. 2a: *T. cruzi*-infected individuals. 2b: Uninfected individuals. Rho values are shown. Significant correlations (p<0.05) are shaded gray (positive correlation) and black (negative correlation).**

| **2a. Tc+ Individuals with biomarker levels** | | | | | | | | | | | |
| --- | --- | --- | --- | --- | --- | --- | --- | --- | --- | --- | --- |
|  | **Stage** | **BNP** | **NTproBNP** | **CKMB** | **Troponin I** | **MMP-2** | **MMP-9** | **TGFB1** | **TGFB2** | **TIMP-1** | **TIMP-2** |
| **Stage** | 1.00 |  |  |  |  |  |  |  |  |  |  |
| **BNP** | 0.25 | 1.00 |  |  |  |  |  |  |  |  |  |
| **NTproBNP** | 0.50 | 0.45 | 1.00 |  |  |  |  |  |  |  |  |
| **CKMB** | 0.13 | 0.10 | 0.10 | 1.00 |  |  |  |  |  |  |  |
| **Troponin I** | 0.24 | 0.21 | 0.26 | 0.10 | 1.00 |  |  |  |  |  |  |
| **MMP-2** | 0.29 | 0.30 | 0.37 | 0.20 | -0.04 | 1.00 |  |  |  |  |  |
| **MMP-9** | -0.08 | -0.02 | -0.11 | 0.03 | -0.04 | -0.20 | 1.00 |  |  |  |  |
| **TGFB1** | -0.14 | -0.06 | -0.15 | -0.03 | -0.07 | -0.25 | 0.25 | 1.00 |  |  |  |
| **TGFB2** | -0.04 | -0.04 | -0.12 | -0.01 | 0.09 | -0.08 | 0.12 | 0.57 | 1.00 |  |  |
| **TIMP-1** | 0.18 | 0.08 | 0.19 | -0.01 | 0.04 | 0.30 | -0.25 | 0.04 | 0.11 | 1.00 |  |
| **TIMP-2** | 0.20 | 0.12 | 0.27 | 0.00 | 0.01 | 0.58 | 0.03 | -0.11 | 0.17 | 0.66 | 1.00 |
|  |  |  |  |  |  |  |  |  |  |  |  |
| **2b. Tc- Individuals with biomarker levels** | | | | | | | | | | | |
|  | **Stage** | **BNP** | **NTproBNP** | **CKMB** | **Troponin I** | **MMP-2** | **MMP-9** | **TGFB1** | **TGFB2** | **TIMP-1** | **TIMP-2** |
| **Stage** | 1.00 |  |  |  |  |  |  |  |  |  |  |
| **BNP** | 0.25 | 1.00 |  |  |  |  |  |  |  |  |  |
| **NTproBNP** | 0.44 | 0.16 | 1.00 |  |  |  |  |  |  |  |  |
| **CKMB** | 0.27 | -0.04 | 0.21 | 1.00 |  |  |  |  |  |  |  |
| **Troponin I** | 0.30 | 0.19 | 0.37 | 0.08 | 1.00 |  |  |  |  |  |  |
| **MMP-2** | 0.23 | 0.16 | 0.45 | 0.03 | 0.09 | 1.00 |  |  |  |  |  |
| **MMP-9** | -0.01 | 0.09 | 0.03 | 0.08 | 0.04 | -0.17 | 1.00 |  |  |  |  |
| **TGFB1** | -0.24 | -0.04 | -0.17 | -0.33 | -0.24 | -0.18 | -0.08 | 1.00 |  |  |  |
| **TGFB2** | -0.27 | 0.00 | -0.14 | -0.18 | -0.21 | -0.07 | 0.06 | 0.68 | 1.00 |  |  |
| **TIMP-1** | 0.00 | 0.16 | 0.13 | -0.18 | -0.01 | 0.30 | -0.26 | -0.03 | -0.04 | 1.00 |  |
| **TIMP-2** | -0.02 | 0.20 | 0.07 | -0.06 | -0.14 | 0.43 | 0.03 | -0.14 | 0.10 | 0.67 | 1.00 |

Rho values are shown. Significant correlations are shaded gray (positive correlation) and black (negative correlation) for p<0.05.
