# Supplementary material for: Biomarkers in Trypanosoma cruzi-Infected and Uninfected Individuals with Varying Severity of Cardiomyopathy in Santa Cruz, Bolivia
Source: PLoS Negl Trop Dis. 2014 Oct 2;8(10):e3227. doi: 10.1371/journal.pntd.0003227 (PMC4183477; doi:10.1371/journal.pntd.0003227)
Supplement: Figure S3 — Correlation between biomarker levels among T. cruzi-infected individuals stratified by stage. 3a: Stage AB. 3b: Stage CD. Rho values are shown. Significant positive correlations (p<0.05) are shaded gray. No significant negative correlations were observed. (DOCX) [file pntd.0003227.s004.docx]

**Figure S3: Correlation between biomarker levels among *T. cruzi*-infected individuals stratified by stage. 3a: Stage AB. 3b: Stage CD. Rho values are shown. Significant positive correlations (p < 0.05) are shaded gray. No significant negative correlations were observed.**

|  | | | | | | | | | | | | | | | | | | | | | | | | | | | | | | | |  |
| --- | --- | --- | --- | --- | --- | --- | --- | --- | --- | --- | --- | --- | --- | --- | --- | --- | --- | --- | --- | --- | --- | --- | --- | --- | --- | --- | --- | --- | --- | --- | --- | --- |
|  | | **BNP** | | **NTproBNP** | | | | **CKMB** | | | **Troponin I** | | | **MMP-2** | | | **MMP-9** | | | **TGFB1** | | | **TGFB2** | | | **TIMP-1** | | | **TIMP-2** | | | |
| **BNP** | | 1.00 | |  | | | |  | | |  | | |  | | |  | | |  | | |  | | |  | | |  | | | |
| **NTproBNP** | | 0.24 | | 1.00 | | | |  | | |  | | |  | | |  | | |  | | |  | | |  | | |  | | | |
| **CKMB** | | -0.04 | | -0.04 | | | | 1.00 | | |  | | |  | | |  | | |  | | |  | | |  | | |  | | | |
| **Troponin I** | | 0.22 | | 0.00 | | | | 0.07 | | | 1.00 | | |  | | |  | | |  | | |  | | |  | | |  | | | |
| **MMP-2** | | 0.02 | | 0.11 | | | | 0.10 | | | -0.15 | | | 1.00 | | |  | | |  | | |  | | |  | | |  | | | |
| **MMP-9** | | 0.07 | | 0.02 | | | | 0.02 | | | 0.04 | | | -0.08 | | | 1.00 | | |  | | |  | | |  | | |  | | | |
| **TGFB1** | | -0.08 | | -0.07 | | | | -0.01 | | | -0.02 | | | -0.35 | | | 0.23 | | | 1.00 | | |  | | |  | | |  | | | |
| **TGFB2** | | -0.16 | | -0.08 | | | | 0.05 | | | 0.14 | | | -0.12 | | | 0.06 | | | 0.58 | | | 1.00 | | |  | | |  | | | |
| **TIMP-1** | | 0.04 | | 0.03 | | | | -0.08 | | | 0.05 | | | 0.34 | | | -0.22 | | | -0.04 | | | 0.04 | | | 1.00 | | |  | | | |
| **TIMP-2** | | -0.01 | | 0.09 | | | | -0.12 | | | 0.01 | | | 0.61 | | | 0.16 | | | -0.12 | | | 0.17 | | | 0.66 | | | 1.00 | | | |
|  | | |  | |  | |  | | |  | | |  | | |  | | |  | | |  | | |  | | |  | | |  |  |
| **3b. Stage CD.** | | | | | | | | | | | | | | | | | | | | | | | | | | | | | | | |  |
|  | **BNP** | | | | | **NTproBNP** | | | **CKMB** | | | **Troponin I** | | | **MMP-2** | | | **MMP-9** | | | **TGFB1** | | | **TGFB2** | | | **TIMP-1** | | | **TIMP-2** | | |
| **BNP** | 1.00 | | | | |  | | |  | | |  | | |  | | |  | | |  | | |  | | |  | | |  | | |
| **NTproBNP** | 0.61 | | | | | 1.00 | | |  | | |  | | |  | | |  | | |  | | |  | | |  | | |  | | |
| **CKMB** | 0.10 | | | | | 0.21 | | | 1.00 | | |  | | |  | | |  | | |  | | |  | | |  | | |  | | |
| **Troponin I** | 0.06 | | | | | 0.12 | | | 0.17 | | | 1.00 | | |  | | |  | | |  | | |  | | |  | | |  | | |
| **MMP-2** | 0.45 | | | | | 0.58 | | | 0.19 | | | -0.06 | | | 1.00 | | |  | | |  | | |  | | |  | | |  | | |
| **MMP-9** | -0.05 | | | | | -0.13 | | | 0.00 | | | 0.08 | | | -0.33 | | | 1.00 | | |  | | |  | | |  | | |  | | |
| **TGFB1** | -0.07 | | | | | -0.14 | | | 0.01 | | | -0.14 | | | -0.10 | | | 0.32 | | | 1.00 | | |  | | |  | | |  | | |
| **TGFB2** | -0.11 | | | | | -0.18 | | | -0.13 | | | -0.06 | | | -0.08 | | | 0.03 | | | 0.52 | | | 1.00 | | |  | | |  | | |
| **TIMP-1** | 0.06 | | | | | 0.21 | | | 0.04 | | | -0.10 | | | 0.21 | | | -0.28 | | | 0.16 | | | 0.17 | | | 1.00 | | |  | | |
| **TIMP-2** | 0.15 | | | | | 0.30 | | | 0.04 | | | -0.12 | | | 0.50 | | | -0.08 | | | -0.08 | | | 0.13 | | | 0.61 | | | 1.00 | | |

Significant positive correlations (p < 0.05) are shaded gray. No significant negative correlations were observed.
