# Supplementary material for: Biomarkers in Trypanosoma cruzi-Infected and Uninfected Individuals with Varying Severity of Cardiomyopathy in Santa Cruz, Bolivia
Source: PLoS Negl Trop Dis. 2014 Oct 2;8(10):e3227. doi: 10.1371/journal.pntd.0003227 (PMC4183477; doi:10.1371/journal.pntd.0003227)
Supplement: Table S1 — Basic characteristics and risk factors by T. cruzi infection status in all individuals meeting inclusion criteria. (DOCX) [file pntd.0003227.s005.docx]

**Table S1: Basic characteristics and risk factors by *T. cruzi*-infection status in all individuals meeting inclusion criteria.**

|  | | **Tc-** (N=99) | **Tc+** (N=310) | **P-value** |
| --- | --- | --- | --- | --- |
|  | | N (%) | N (%) |  |
| Age in years, median (SD) | | 56.0 (13.9) | 58.2 (12.3) | 0.13 |
| Male | | 42 (42.4) | 158 (51.0) | 0.14 |
| Weight in kg, median (SD)* | | 71.1 (16.5) | 72.0 (15.3) | 0.63 |
| BMI in kg/m^2^* | | 27.7 (5.5) | 28.4 (5.5) | 0.31 |
|  | Underweight (BMI<18.5)* | 3 (3.7) | 3 (1.0) | 0.10 |
|  | Normal weight (BMI 18.5-25)* | 20 (24.4) | 76 (26.3) | 0.73 |
|  | Overweight (BMI 25-30)* | 32 (39.0) | 105 (36.3) | 0.66 |
|  | Obese (BMI>=30)* | 27 (32.9) | 105 (36.3) | 0.57 |
| Self-reported medical history | |  |  |  |
|  | Hypertension | 42 (42.4) | 138 (44.5) | 0.72 |
|  | Diabetes | 14 (14.1) | 39 (12.6) | 0.69 |
|  | Coronary artery disease | 9 (9.1) | 34 (11.0) | 0.60 |
| Chagas-associated risk factors | |  |  |  |
|  | Ever lived in rural area | 66 (66.7) | 254 (81.9) | **<0.01** |
|  | Ever seen triatomine in home | 54 (54.6) | 276 (89.0) | **<0.01** |
|  | Ever lived in a home with mud walls | 53 (53.5) | 256 (82.6) | **<0.01** |
|  | Ever lived in a home with thatched roof | 29 (29.3) | 124 (40.0) | 0.06 |
|  | Family history of Chagas disease | 26 (26.3) | 165 (53.2) | **<0.01** |
| Indicators of socioeconomic status | |  |  |  |
|  | Electricity | 98 (99.0) | 301 (97.1) | 0.29 |
|  | Refrigerator | 87 (87.9) | 259 (83.6) | 0.30 |
|  | Cellphone | 89 (89.9) | 286 (86.8) | 0.41 |
|  | Attended some high school | 44 (44.4) | 77 (24.8) | **<0.01** |

*Not all individuals had weight/BMI information, for Tc- N=82, for Tc+ N=289.
