# Supplementary material for: Biomarkers in Trypanosoma cruzi-Infected and Uninfected Individuals with Varying Severity of Cardiomyopathy in Santa Cruz, Bolivia
Source: PLoS Negl Trop Dis. 2014 Oct 2;8(10):e3227. doi: 10.1371/journal.pntd.0003227 (PMC4183477; doi:10.1371/journal.pntd.0003227)
Supplement: Table S2 — ECG findings suggestive of Chagas disease by T. cruzi infection status for individuals included in biomarker analysis. (DOCX) [file pntd.0003227.s006.docx]

**Table S2: ECG findings suggestive of Chagas disease by *T. cruzi*-infection status for individuals included in biomarker analysis.**

| **ECG Finding** | **Tc-** | **Tc+** | **P-value** |
| --- | --- | --- | --- |
|  | **N=72** | **N=193** |  |
| Bradycardia (<=50 bpm) | 0 (0) | 15 (7.8) | **0.01** |
| Atrial fibrillation or atrial flutter | 4 (5.6) | 29 (15.0) | **0.04** |
| Multiform PVCs or bigeminy | 1 (1.4) | 7 (3.6) | 0.69 |
| 1st degree AVB | 4 (5.6) | 28 (14.5) | 0.06 |
| 3rd degree AVB | 1 (1.4) | 4 (2.1) | 1.00 |
| Incomplete RBBB | 2 (2.8) | 11 (5.7) | 0.52 |
| RBBB only | 3 (4.2) | 22 (11.4) | 0.10 |
| LBBB | 2 (2.8) | 12 (6.2) | 0.36 |
| LAFB only | 5 (6.9) | 11 (5.7) | 0.71 |
| LPFB | 0 (0) | 0 (0) | Na |
| Bifascicular block (RBBB and LAFB) | 1 (1.4) | 9 (4.7) | 0.30 |
| NIVCD | 5 (6.9) | 32 (16.6) | **0.04** |
| Any complete BBB (RBBB, LBBB, LAFB, LPFB) | 11 (15.3) | 54 (28.0) | **0.03** |

bpm = beats per minute, PVC = premature ventricular contractions, AVB = atrioventricular block, RBBB = right bundle branch block, LBBB = left bundle branch block, LAFB = left anterior fascicular block, LPFB = left posterior fascicular block, NIVCD = nonspecific intraventricular conduction delay, BBB bundle branch block, na = not applicable
