# Supplementary material for: Biomarkers in Trypanosoma cruzi-Infected and Uninfected Individuals with Varying Severity of Cardiomyopathy in Santa Cruz, Bolivia
Source: PLoS Negl Trop Dis. 2014 Oct 2;8(10):e3227. doi: 10.1371/journal.pntd.0003227 (PMC4183477; doi:10.1371/journal.pntd.0003227)
Supplement: Table S3 — Comparisons of biomarkers and biomarker ratios by stage within T. cruzi-infected (Tc+) and uninfected (Tc−) groups. (DOCX) [file pntd.0003227.s007.docx]

**Table S3: Comparisons of biomarkers and biomarker ratios by stage within *T. cruzi*-infected (Tc+) and uninfected (Tc-) groups.**

|  | **Comparison within Tc+ group**  **P values** | | | | **Comparisons within Tc- group**  **P values** | | |
| --- | --- | --- | --- | --- | --- | --- | --- |
|  | **A vs B** | **A vs CD** | **B**  **vs CD** | **AB vs CD** | **A vs B** | **A**  **vs CD** | **AB**  **vs CD** |
| **Biomarker (pg/ml)** |  |  |  |  |  |  |  |
| BNP | 0.79 | **<0.01** | **<0.01** | **<0.01** | 0.27 | 0.07 | 0.10 |
| NTproBNP | 0.94 | **<0.01** | **<0.01** | **<0.01** | 0.051 | **<0.01** | **<0.01** |
| CKMB | 0.75 | 0.11 | 0.30 | 0.10 | 0.96 | **0.01** | **0.01** |
| Troponin I | 0.32 | **<0.01** | 0.06 | **<0.01** | 0.29 | **0.02** | **0.04** |
| MMP-2 | 0.34 | **<0.01** | **0.03** | **<0.01** | 0.06 | 0.17 | 0.26 |
| MMP-9 | 0.23 | 0.16 | 0.99 | 0.32 | 0.56 | 0.56 | 0.55 |
| TIMP-1 | 0.64 | **0.04** | **0.01** | **<0.01** | 0.27 | 0.91 | 0.90 |
| TIMP-2 | 0.68 | **0.04** | **0.01** | **<0.01** | 0.58 | 0.72 | 0.62 |
| TGFB1 | 0.35 | 0.11 | 0.63 | 0.17 | 1.00 | 0.08 | 0.11 |
| TGFB2 | 0.93 | 0.85 | 0.72 | 0.76 | 0.54 | **0.02** | **0.02** |
| **Biomarker Ratios** |  |  |  |  |  |  |  |
| MMP-2/MMP-9 | 0.07 | **<0.01** | 0.24 | **<0.01** | 0.71 | 0.39 | 0.39 |
| MMP-2/TGFB1 | 0.28 | **<0.01** | 0.07 | **<0.01** | 0.08 | 0.14 | 0.20 |
| MMP-2/TIMP-2 | 0.22 | 0.15 | 0.92 | 0.31 | 0.74 | 0.07 | 0.08 |
| MMP-9/TIMP-1 | 0.21 | **0.05** | 0.36 | 0.07 | 0.98 | 0.56 | 0.56 |

The number (N) in each group is listed in Table 2. P-value refers to comparison between the two groups listed.
